# Magnesium Excretion in *C. elegans* Requires the Activity of the GTL-2 TRPM Channel

**DOI:** 10.1371/journal.pone.0009589

**Published:** 2010-03-08

**Authors:** Takayuki Teramoto, Laura A. Sternick, Eriko Kage-Nakadai, Shirine Sajjadi, Jakub Siembida, Shohei Mitani, Kouichi Iwasaki, Eric J. Lambie

**Affiliations:** 1 Department of Biology, Kyushu University, Higashi-ku, Fukuoka, Japan; 2 Department of Biological Sciences, Dartmouth College, Hanover, New Hampshire, United States of America; 3 Department of Molecular Pharmacology & Biological Chemistry, Northwestern University Feinberg School of Medicine, Chicago, Illinois, United States of America; 4 Department of Physiology, Tokyo Women's Medical University School of Medicine, Tokyo, Japan; Brown University, United States of America

## Abstract

Systemic magnesium homeostasis in mammals is primarily governed by the activities of the TRPM6 and TRPM7 cation channels, which mediate both uptake by the intestinal epithelial cells and reabsorption by the distal convoluted tubule cells in the kidney. In the nematode, *C. elegans*, intestinal magnesium uptake is dependent on the activities of the TRPM channel proteins, GON-2 and GTL-1. In this paper we provide evidence that another member of the TRPM protein family, GTL-2, acts within the *C. elegans* excretory cell to mediate the excretion of excess magnesium. Thus, the activity of GTL-2 balances the activities of the paralogous TRPM channel proteins, GON-2 and GTL-1.

## Introduction

In humans, the bulk of systemic magnesium uptake occurs via the small intestine and is mediated by the action of the TRPM6 (and probably TRPM7) cation channels (reviewed in [1], [2]). Mutations in the human TRPM6 gene result in hereditary hypomagnesemia [3], [4], [5]. A paracellular magnesium uptake mechanism is also thought to exist within the mammalian intestine, but it has not been well characterized [6], [7], [8]. In the nematode *C. elegans*, intestinal Mg^2+^ uptake requires the redundant activities of the paralogous GON-2 and GTL-1 TRPM channel proteins [9]; however, no paracellular pathway has been described.

In mammals, circulating levels of magnesium are maintained within a fairly narrow range [10], and this is dependent on the activity of TRPM6 (and probably TRPM7) within the distal convoluted tubule of the kidney [3], [4], [5]. In this context, these channels function by mediating reabsorption of magnesium from the filtrate. In *C. elegans*, the mechanisms that govern magnesium excretion have not been characterized. However, we previously reported that the *gtl-2* gene (paralogous to *gon-2* and *gtl-1*) is expressed within the excretory cell [9], which is thought to perform physiological functions comparable to those of the mammalian kidney. In this paper we show that the action of GTL-2 within the excretory cell is necessary for normal Mg^2+^ excretion. GTL-2 localizes to the basal membrane of the excretory cell, suggesting that it mediates uptake of Mg^2+^ from the pseudocoelomic fluid.

## Results

### Identification of Mutations in *gtl-2*

**Figure 1:**
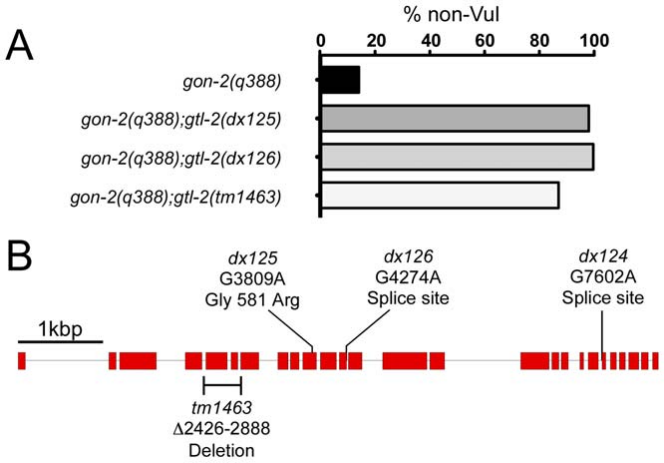
Suppression of *gon-2(q388)* by *gtl-2* mutations. **A.** Efficiency of suppression. Animals were raised at the restrictive temperature for *gon-2(q388)* and scored as adults for the presence of the vulva, which provides an accurate readout of the successful initiation of gonadogenesis. **B.** Nature and locations of *gtl-2* alleles. Boxes and lines indicate exons and introns, respectively. *dx124* and *dx126* are splice-site mutations, and *dx125* converts glycine to arginine at the 581st amino acid. The *gtl-2(tm1463)* mutation was independently isolated by the *C. elegans* genome knockout project in Japan. *gtl-2(tm1463)* corresponds to a deletion of 463 base pairs, from the fifth through seventh exons of *gtl-2*. 5′ end of *gtl-2* is at left.

As detailed in the materials and methods section, we selected for suppressors of the sterile phenotype of the temperature-sensitive allele, *gon-2(q388)*, using procedures similar to those reported previously [11], [12]. Three of the mutations that we identified in this screen appeared to be alleles of the same gene, since they all mapped to the gene cluster on chromosome IV, failed to complement each other and exhibited a similar combination of phenotypes, i.e., suppression of *gon-2(q388)* (Figure 1A), slow growth (Gro), and egg-laying-defective (Egl). We recognized the latter two phenotypes as similar to those of a deletion/candidate null allele of the *gtl-2* locus (*tm1463*), which also maps to the chromosome IV gene cluster. Consistent with the possibility that our suppressor mutations are alleles of *gtl-2*, each of them failed to complement *tm1463*. Furthermore, we found that *gtl-2(tm1463)* is also able to suppresses *gon-2(q388)* (Figure 1A). To further verify allelism, we sequenced the *gtl-2* exonic regions for each of the suppressor mutations. In each case, we found an alteration that has the potential to impair GTL-2 function (Figure 1B).

### Expression and Localization of GTL-2

**Figure 2:**
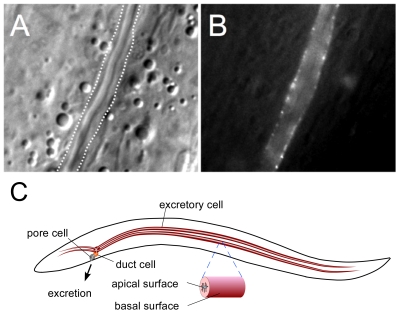
Subcellular localization of GTL-2. A. DIC image, with dashed line indicating position of basal membrane of excretory cell as determined by imaging in GFP channel. B. GFP fluorescence image. The imaged animal was of genotype *gtl-2(tm1463);tgEx133[Pgtl-2::gtl-2cDNA::gfp;Pgtl-2::mCherry]*. mCherry fluorescence completely fills the space between the canal lumen and the basal membrane (see Figure S1). C. Schematic of excretory system of *C. elegans*. The excretory system comprises three cell types (excluding the gland cells): a single large excretory cell, a duct cell, and a pore cell. The pore cell connects the duct to the main body hypodermis, and a binucleate gland cell, the fourth cell, bridges between the excretory cell and the duct cell [37]. The excretory cell is H-shaped and it extends two arms along the lateral lines on each side of the animal body. The excretory cell is polarized with basal and apical faces. The apical face is adjacent to a lumen, which is connected to the outside of the body through the excretory pore. The basal side faces the pseudocoelomic space. It is believed that the excretory cell absorbs excess electrolytes from the pseudocoelomic fluid, then transfers them to the lumen, followed by their excretion to the outer environment through the excretory pore.

We previously found that a *gtl-2* transcriptional reporter was expressed in the excretory cell [9]. In order to determine the subcellular localization of GTL-2 protein, we generated a GTL-2::GFP translation fusion construct and expressed this in *C. elegans* under the control of the *gtl-2* promoter. The GTL-2::GFP fusion protein is predominantly localized to the outer/basal surface of the excretory cell (Figure 2, Figure S1). The punctate expression of GTL-2::GFP is similar to that observed for TRPM6 and TRPM7 in cultured cells [13]. The subcellular localization of GTL-2 is clearly different from proteins such as EXC-4, which are present on the apical surface of the excretory cell [14]. The basal membrane is likely to be the normal site of action for GTL-2, because this fusion protein fully rescues the *gtl-2(tm1463)* mutant phenotype (see below). However, we cannot rule out the possibility that a small fraction of GTL-2 is also expressed on the apical surface of the excretory cell.

To further confirm that the functional site of *gtl-2* is the excretory cell, we used the *sulp-4* promoter to drive expression of the *gtl-2::gfp* fusion gene. *sulp-4* encodes one of eight members of the sulfate permease family of anion transporters, and its promoter drives GFP expression specifically in the excretory cell [15]. This fusion gene behaved essentially the same as the one driven by the *gtl-2* promoter (data not shown).

### Electrophysiological Phenotype of *gtl-2* Mutants

**Figure 3:**
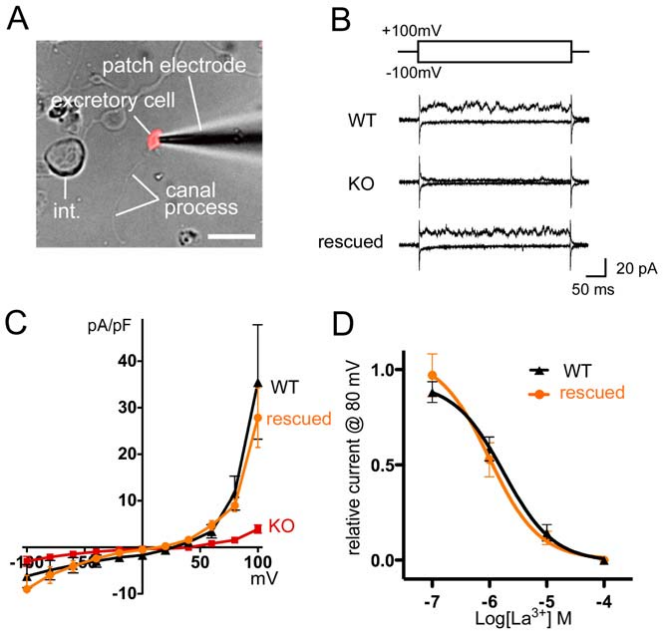
GTL-2 is responsible for the outwardly-rectifying current of the excretory cell. (A) A cultured excretory cell. The bar at the bottom right is 5 microns. The main body of the excretory cell and a straight, unbranched canal process are indicated. “int.” is an intestinal cell. (B) Representative whole-cell current at −100 mV and +100 mV pulses of the excretory cells isolated from the wild-type (WT), *gtl-2(tm1463)* mutant (KO), and a rescued transgenic line (rescued) of *gtl-2(tm1463):tgEx135[Psupl-4::gtl-2cDNA::gfp;Psulp-4::mCherry]*. C) Steady-state I-V relationship for leak-subtracted whole-cell currents (n = 4−6 each, the bars indicate SEM.). (D) Dose-dependent inhibition of the whole-cell current by La^3+^ (n = 4 each point, the bars indicate SEM).

If GTL-2 forms a channel with properties similar to those of other TRPM family members, then the excretory cell should exhibit characteristic outward rectification [16]. In wild type excretory cells, we observed a small outwardly-rectifying current using the whole-cell configuration (its reversal potential [E_rev_] = 26.9+/−1.8 mV in Figure 3A and C); this current appears to be voltage-modulated, which is also observed with TRPM4b and TRPM5 as well as with GON-2 [17] (see Materials and Methods for details). In *gtl-2* mutant cells, we found that the outwardly-rectifying current was eliminated (Figure 3B and C). When the GTL-2 cDNA was expressed in the excretory cell of the *gtl-2* mutants, the outwardly-rectifying current was restored, suggesting that GTL-2 expression is responsible for current generation (Figure 3B and C).

Since TRPM channel activities are known to be inhibited by La^3+^ [18], we tested whether the outwardly-rectifying current of the excretory cell can be inhibited by La^3+^. The current was indeed suppressed nearly completely by 100 µM LaCl_3_ in the bath solution; IC50 values of La^3+^ were 1.7+/−0.5 µM in the wild type and 1.0+/−0.5 µM in the rescued strain (Figure 3D).

Since obtaining recordings from the excretory cell was technically difficult, we were not able to determine the relative permeability of GTL-2 to different cations; however, given the high degree of similarity between GTL-2 and GTL-1/GON-2, which are known to be permeable to both Ca^2+^ and Mg^2+^ [9], [19], [20], [21], it is likely that GTL-2 is also permeable to these cations.

### Effects of Mg^2+^ Supplementation on *gon-2* and *gtl-2* Mutants

We previously reported that supplementation of the culture medium with Mg^2+^, but not Ca^2+^, can suppress the growth defect of the *gon-2*; *gtl-1* double mutant [9]. In this study, we found that Mg^2+^ supplementation can also partially suppress the gonadogenesis defect of *gon-2* mutants to a degree that approaches that observed for the *gtl-2* mutations; supplementation of the medium with 50 mM Mg^2+^ increased the frequency of non-vulvaless *gon-2(q388)* animals raised at 23.5° from 12% (n = 763) to 64% (n = 968).

**Figure 4:**
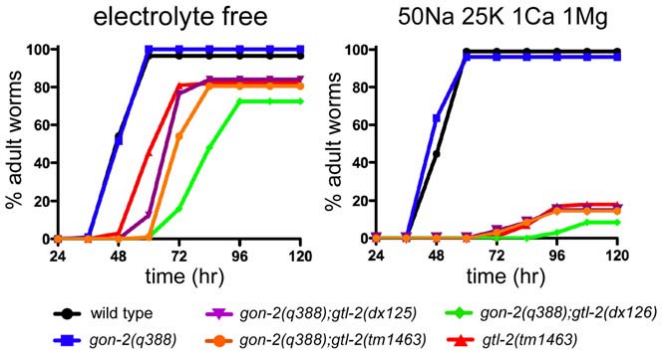
Growth inhibitory effects of Mg^2+^ on *gtl-2* mutants isolated as suppressors of *gon-2(q388)*. Assays were performed as described in Methods. The means of two independent results are plotted. The X-axis is time (hours) after hatching and the Y-axis is the percentage of the adult animals over total animals. All *gtl-2* mutants showed slower growth than wild type, which was more pronounced in the presence of ions (right panel), compared to the electrolyte-free condition (left panel). Ion concentrations are in mM.

**Figure 5:**
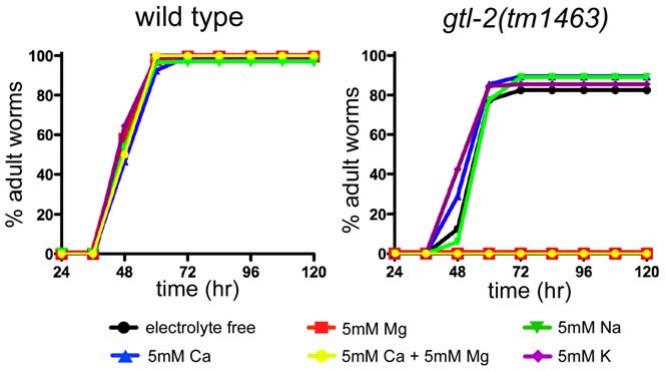
Effects of different cations on growth rate of the putative null mutant, *gtl-2(tm1463)*. Magnesium specifically inhibits the growth of *gtl-2(tm1463)* mutant animals. Assays were performed as described in Methods. The means of two independent results are plotted. As indicated, 5 mM NaCl, 5 mM KCl, 5 mM MgCl_2_, and 5 mM CaCl_2_ were included.

In stark contrast to the results obtained with *gon-2* and *gtl-1*, supplementation with Mg^2+^, but not other cations (Ca^2+^, Na^+^, and K^+^), greatly enhances the *gtl-2* mutant phenotype, and at higher concentrations results in larval arrest and lethality (Figures 4 and 5). Together with the expression data, these results suggest that mutations in *gtl-2* impair uptake of Mg^2+^ by the excretory cell, and that suppression of *gon-2(q388)* results from a buildup of Mg^2+^ within the pseudocoelomic fluid.

### Suppression of *gtl-2(lf)* by Mutations in *gtl-1*

**Figure 6:**
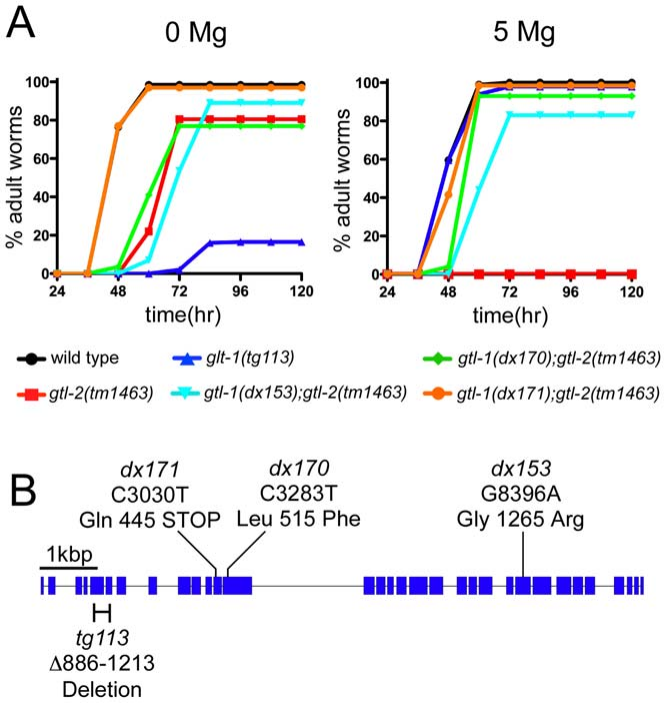
Characterization of *gtl-1* alleles isolated as suppressors of *gtl-2(tm1463)*. A. Effects of Mg^2+^ on growth rate of *gtl-1* and *gtl-2* single and double mutants.Assays were performed as described in Methods, using either 0 mM or 5 mM Mg^2+^ supplementation. The means of two independent results are plotted. The *gtl-1(tg113)-*single mutant shows growth defects on 0 mM Mg^2+^ plates as previously reported [9]. B. Locations of mutant alleles within the *gtl-1* locus. The *gtl-1* gene and sites of the *gtl-2* suppressor mutations. Boxes and lines indicate exons and introns, respectively. The *tg113* allele deletes nucleotides 886-1213. 5′ end of *gtl-1* is at left.

If mutations in *gtl-2* cause impairment of growth due to a defect in the excretion of Mg^2+^, it should be possible to remedy this growth defect by reducing the amount of Mg^2+^ absorbed by the gut. A simple way to test this idea would be to determine whether impairment of intestinal Mg^2+^ uptake by mutation of *gtl-1* can suppress the Gro phenotype of *gtl-2*. However, the proximity of the *gtl-1* and *gtl-2* loci (13 kbp) makes it very difficult to obtain the double mutant by standard strain construction methods. Therefore, as an alternative approach, we performed a random mutagenesis and directly selected for mutations that could suppress the lethality of *gtl-2(tm1463)* on 50 mM Mg^2+^. We characterized six independently-isolated suppressors obtained from this selection. The suppression phenotype for three of these is shown in Figure 6A. Each of the six suppressors failed to segregate Gtl-2 progeny after backcrossing, suggesting tight linkage to *tm1463*, and thus consistent with the possibility that a mutation had occurred in *gtl-1*. We sequenced the *gtl-1* coding sequence for all six suppressors and found a single alteration in each case (Figure 6B). Two of these are splice site mutations and two are premature stop codons, strongly suggesting that these are severe loss-of-function mutations.

As a further test, we performed RNAi knock-down of *gtl-1* in a *gtl-2(tm1463)* mutant background. In these experiments, we found that *gtl-1* RNAi treatment produced a phenotype extremely similar to that of the suppressor mutations described above (Figure S2).

### Assessment of Magnesium Levels by ICP-MS

We used ICP-MS to compare the total Mg^2+^ content in wild type animals and animals of genotype *gtl-2(tm1463)* in one experiment, and wild type animals and *gtl-1(dx153,dx170,dx171) gtl-2(tm1463)* in a separate experiment. In the first experiment, we found that *gtl-2(tm1463)* animals contain approximately 1.5 times as much Mg^2+^ per unit mass as wild type animals (Table 1). In the second experiment, we found that *gtl-1;gtl-2* double mutants contain approximately 0.7 times as much Mg^2+^ as wild type animals (Table 2). Notably, these animals are not as severely affected as *gtl-1* single mutants, which contain only approximately 0.4 times as much Mg^2+^ as wild type [9]. This suggests that mutations in *gtl-1* and *gtl-2* exert mutual suppression.

**Table 1:** Trace elements in wild type and *gtl-2(tm1463)* mutant animals.

Genotype	Mg (p = 0.001)	K (p = 0.18)	Ca (p = 0.0082)	n
wild type	2022+/−10	15854+/−802	2976+/−119	3
*gtl-2(tm1463)*	3035+/−202	14277+/−1462	1991+/−329	3

Levels of each trace element are in µg/g, ± standard deviation. ICPMS analysis was performed as described in materials and methods. p values were determined using a two-tailed unpaired t-test.

**Table 2:** Trace elements measured in wild type and *gtl-1 gtl-2* double mutants.

Genotype	Mg	K	n
wild type	2141+/−9	15652+/−809	2
*gtl-1(dx153) gtl-2(tm1463)*	1584+/−92 P = 0.013	15494+/−245 P = 0.8163	2
*gtl-1(dx170) gtl-2(tm1463)*	1564+/−14 P = 0.0004	15182+/−14 P = 0.4977	2
*gtl-1(dx171) gtl-2(tm1463)*	1499+/−215 P = 0.0507	14819+/−880 P = 0.4283	2

In each case, values represent the average µg/g for animals grown on 0 mM and 1 mM supplemental Mg^2+^. Values for Ca^2+^ are reported in Table S1, because they were strongly affected by Mg^2+^ supplementation. P values were calculated as in Table 1.

Although potassium levels were not affected by mutation of *gtl-2*, Ca^2+^ levels were significantly reduced, about 0.7 times wild type levels. This may be due to inhibition of GON-2, since this channel is highly sensitive to Mg^2+^ levels and also known to be important for Ca^2+^uptake by the intestinal cells [9], [20]. This idea is consistent with the observation that increasing levels of Mg^2+^in the medium tends to cause a decrease in total Ca^2+^ concentration in both wild type animals and animals of genotype *gtl-1(lf) gtl-2(tm1463)* (Table S1).

## Discussion

**Figure 7:**
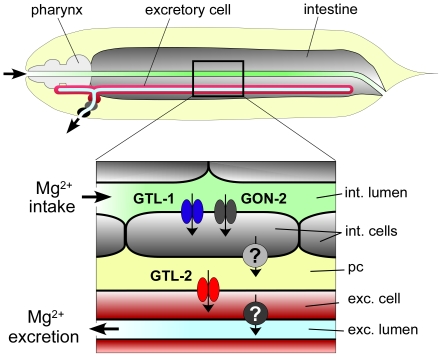
Schematic of proposed roles for *C. elegans* TRPM channels in Mg^2+^ uptake and excretion. Details discussed in text. pc, pseudocoelom.

In this paper, we demonstrate that inactivation of the *gtl-2* gene of *C. elegans* results in suppression of the *gon-2* mutant phenotype and also causes hypermagnesemia. Furthermore, we find that inactivation of *gtl-1* suppresses the hypermagnesemic phenotype of *gtl-2* mutants. In combination with findings from previous studies, we suggest the following model to explain these results (Figure 7). Mg^2+^ within the gut lumen is taken up by the intestinal cells through apically-localized channels composed of GTL-1 and/or GON-2 subunits [9]. These channels are equivalent to IORCa, as described by Strange and colleagues [20], [21], [22]. Mg^2+^ is transported out of the intestinal cells and into the pseuodocoelomic fluid through an unknown mechanism (possibly via a Na^+^/Mg^2+^ antiporter). Mg^2+^ within the pseuodocoelomic fluid bathes all of the exposed tissues within the body cavity, including the gonadal precursor cells (not shown). The gonadal precursors take up Mg^2+^ via two pathways, one mediated by GON-2 and the other dependent on the monocarboxylate transporter family protein, GEM-1 [12]. The excretory cell expresses GTL-2 on its basal membrane, and this permits uptake of Mg^2+^ into the cytoplasm. Mg^2+^ is then transported into the canal lumen via an unknown mechanism.

Our model explicitly requires that Mg^2+^ uptake via the intestinal cells is mediated by TRPM channels. However, Jin *et al.* [23] have recently argued that TRPM channels in mammals are not capable of conducting Mg^2+^ at a rate sufficiently high to provide a viable Mg^2+^ uptake system under physiological conditions. This argument is based largely on their finding that knocking out TRPM7 in lymphocytes does not prevent these cells from taking up Mg^2+^ or from proliferating. However, it is known that mammalian cells possess alternative systems for Mg^2+^ uptake (e.g., the SLC41 transporters [24], [25], [26], [27]), so this result is not entirely unexpected. Jin et al. also used electrophysiological and imaging methods to examine TRPM7 mutant cells for defects in acute Mg^2+^ influx, found no apparent defects, and concluded that Mg^2+^ uptake was unaffected. Their conclusion is similar to that of Xing et al. (2008), who suggested that since GON-2 and GTL-1 are approximately an order of magnitude more permeable to Ca^2+^ than Mg^2+^, they are not likely to mediate significant levels of Mg^2+^ uptake in vivo.

In the case of *C. elegans* two questions need to be answered in order to address these concerns:

1. How much Mg^2+^ does a worm need to absorb per hour?
  An adult hermaphrodite produces approximately 4 eggs per hour, each with a volume of approximately 3×10^−11^ liters and an estimated total Mg^2+^ concentration of ∼2×10^−2^ moles/liter (Table 1, assuming that Mg^2+^ concentration in eggs is the same as overall and that *C. elegans* dry weight is 20% wet weight). This would require the absorption of a minimum of 2.4×10^−12^ moles/hr. The amount of Mg^2+^ excreted by a hermaphrodite per hour is unknown; however, a generous estimate would be that it is roughly equivalent to the amount that is provided to eggs, which leads to an estimated total required absorption rate of 4.8×10^−12^ moles/hr/hermaphrodite.
2. Can GON-2/GTL-1 realistically mediate this rate of absorption?
  The number of channels required to mediate this rate of uptake depends on their individual efficiency of ion conduction, which has the potential to vary over many orders of magnitude, ranging from 6×10^−14^ moles/hr (for an efficient channel) to 6×10^−19^ moles/hr (for an ion pump) [28]. Therefore, even if the *C. elegans* TRPM channels operate on the average at only 0.1% of the efficiency of a “respectable” ion channel, only 80,000 channels would be required along the length of the entire intestinal lumen. The dimensions of the gut lumen are approximately 5×10^4^ µm^2^, and the brush border adds another 10-fold increase in surface area (WormAtlas), resulting in an estimate of 5×10^5^ µm^2^ per intestine. Consequently, even for an extremely inefficient ion channel, the density would only have to be 1 per 6.25 µm^2^ in order to support physiological levels of Mg^2+^ uptake, which is far below the likely channel density.

An alternative way of approaching question number two is to compare the relative permeabilities of GON-2/GTL-1 to Ca^2+^ and Mg^2+^. If the channel is only 1/10 as permeable to Mg^2+^ as Ca^2+^, then it is essentially operating at 10% efficiency. Furthermore, due to the periodicity of the defecation cycle, the channel is only open approximately 5% of the time [21], [22], [29]. Even so, this still results in a channel that operates with an overall efficiency of 0.5% optimal, which would be more than sufficient to mediate physiological levels of Mg^2+^ uptake.

Another factor that is likely to be very important is the physical structure of the gut lining. The space between microvilli is sufficiently restricted that it will represent a barrier to free diffusion within the lumenal space. This has been demonstrated to be an important factor in intestinal absorption rates within the mammalian intestine (for villi, rather than microvilli) [30]. Thus, during the defecation cycle of *C. elegans*, the effective Ca^2+^ concentration within the intervillar space is likely to decrease rapidly after channel opening, and this will permit a progressive increase in Mg^2+^ influx.

The increase in Mg^2+^ in *gtl-2* mutants relative to wild type (∼1.4-fold) appears comparatively modest; however, we expect that the majority of the excess Mg^2+^ will be present within the pseudocoelomic fluid, which constitutes a relatively small compartment. If the volume of the pseudocoelom is estimated as 10% of the total animal, then a 1.4-fold overall increase in Mg^2+^ would be equivalent to a 5-fold increase in concentration within the pseudocoelom. Since Mg^2+^ is known to be an effective blocker of certain Ca^2+^ channels [31], [32], this could have a generally deleterious effect on neurotransmission. *gtl-2* mutants do not lay eggs in response to exogenous serotonin (data not shown), consistent with the idea that the egg-laying muscles have diminished sensitivity to neurotransmitter.

## Materials and Methods

### *C. elegans* Strains

In this study, the following *C. elegans* strains and mutations were used: Bristol N2 wild type, *gon-2(q388)*, *gtl-1(tg113)*, *gtl-2(dx125)*, *and gtl-2(dx126)*, *gtl-2(tm1463)*, *gon-2(q388);gtl-2(dx125)*, *gon-2(q388);gtl-2(dx126)*, *and gon-2(q388);gtl-2(tm1463)*, *gtl-1(dx153);gtl-2(tm1463)*, *gtl-1(dx170);gtl-2(tm1463)*, *gtl-1(dx171);gtl-2(tm1463)*, *gtl-1(dx172);gtl-2(tm1463)*, *gtl-1(dx173);gtl-2(tm1463)*, and *gtl-1(dx174);gtl-2(tm1463)*. Mutant strains were typically outcrossed to wild-type males at least three times to reduce background mutations. Since *gtl-2*-single and *gon-2;gtl-2*-double mutant strains exhibit growth defects on conventional NGM (nematode growth medium) -lite agar plates [2.6% (w/v) Bact-Agar (BD), 0.25% (w/v) Bacto-Pepton (BD), 5 mg/L cholesterol (Sigma), 50 mM NaCl, 1 mM MgSO_4_ and 1 mM CaCl_2_, 25 mM KPO_4_ buffer, pH 6.0], we developed new electrolyte-free agar plates [2.6% (w/v) Bact-Agar (BD), 0.25% (w/v) Bacto-Pepton (BD), 5 mg/L cholesterol (Sigma), 100 mM Sucrose (Sigma), and 25 mM Hepes (Sigma), pH 6.0] for improvement of growth and fertility of the mutant strains. Other strains were kept on conventional NGM-lite agar plates at 20°C. Cultured OP50 *E.coli* was used as a food source for the animals, except for strains grown on NGM-lite, which were propagated on AMA1004.

### Isolation of *gon-2(q388)* Suppressors

Adult hermaphrodites of genotype *gon-2(q388) unc-29(e1072)* were mutagenized for 4 hrs with 50 mM EMS at 20°C, then washed and plated onto 120 mm plates at a density of ∼20 hermaphrodites per plate at 15°C (permissive temperature for *gon-2(q388)*). Approximately 1000 F1s reached adult on each plate and each produced approximately 20 F2 progeny before food was depleted on the plate. Half of the animals on each plate were transferred to a large (60 mm) NGM-lite plate enriched with 6X tryptone, and with food source. Then, they were incubated at 25°C (fully restrictive temperature for *gon-2(q388)*) for 10 days. Since food was still present on all plates after 10 days, they were transferred to 19.5°C for suppressor candidates to produce progeny. Individual fertile animals were then cloned from each plate and tested for suppression at 23.5°C (a slightly less restrictive temperature for *gon-2(q388)*). These suppressor candidates were outcrossed to *gon-2(q388)* I*; him-9(e1489)* IV males to assess linkage to *unc-29* and *him-8*. Three independent, non-complementing suppressor mutations were identified in this screen, *dx124*, *dx125* and *dx126*. Each is recessive, linked to *him-8*, and exhibits Egl and Gro secondary phenotypes. The isolation frequency was somewhat lower than expected for mutations in a typical gene (3/240,000 mutagenized genomes, compared to 1/2000 for a typical gene). However, the slow growth of the homozygous mutants probably reduces the efficiency with which they are recovered in suppressor screens.

Suppression of *gon-2(q388)* by *gtl-2* mutations was assessed by scoring for the presence/absence of the vulva via dissecting microscope. The vulva is induced by signals from the gonadal anchor, which is generated after only three cell divisions in the L1 stage. Therefore, the presence of the vulva provides an accurate readout of the completion of initial gonadal cell divisions, which require GON-2 activity. These experiments were done on standard NGM-lite medium.

### Isolation of *gtl-2(tm1463)* Suppressors

*gtl-2(tm1463)* hermaphrodites were mutagenized with 50 mM EMS for 4 hours, then plated in groups on NGM-lite plates at 20°C. F2s and subsequent generations were serially plated on 5 mM MgCl_2_ plates to select for potential suppressors of the *gtl-2(tm1463)* Mg^2+^ toxicity phenotype. Six independently isolated suppressor mutations were chosen for characterization and sequencing.

### Molecular Biology

*C. elegans* genomic DNA was prepared according to Inada et al. [33]. RNA isolation and reverse transcription were performed using RNeasy Mini Kit (Qiagen) and Superscript II reverse transcriptase (Invitrogen). For amplification of genomic DNA and cDNA, LA Taq DNA polymerase (Takara Bio) was used. Amplified DNA fragments were subcloned into pPD95.79 and pBluescript II vectors. DNA sequencing was carried out by the Northwestern University Genomic Core Facility and the Dartmouth Molecular Biology Core Facility.

### Construction of Expression Vectors

The *gtl-2* promoter region used was 2795 bp upstream region from the start codon of the F54D1.5, and the *sulp-4* promoter was 4477 bp upstream from the start codon of the K12G11.1. These promoter fragments were PCR-amplified and subcloned into pPD95.79-mCherry (the original GFP coding region of the pPD95.79 was replaced with mCherry). The final constructs were named *Pgtl-2::mCherry* and *Psulp-4::mCherry*, respectively.

The same promoter regions were used for making the translational GFP-fusion constructs: *Pgtl-2::gtl-2cDNA::gfp and Psulp-4::gtl-2cDNA::gfp*. To prepare the translational reporter constructs, full length *gtl-2* cDNA (4217 bp, without stop codon) was constructed by assembling PCR-amplified cDNA fragments and yk405b5 cDNA (a gift from Y. Kohara) into pPD95.79 vector containing either *Pgtl-2* or *Psupl-4*. After subcloning, the entire cDNAs and junctional regions were sequenced. To construct the ectopic expression vectors, *Pgtl-2::gon-2cDNA*, *Pgtl-2::gtl-1cDNA*, and *Pgtl-2::gtl-2cDNA*, full length cDNAs (including stop codon) of either *gon-2* (5796 bp), *gtl-1* (5085 bp), or *gtl-2* (4421 bp) were prepared using RT-PCR and/or combining yk cDNA clones (yk1643c08 for *gon-2*, yk398a12 and yk440a7 for *gtl-1*, and yk405b5 for *gtl-2*).

### Transgenic Strains

The following transgenic lines were generated and used:

*lin-15(n765ts);tgEx137[Psulp-4::mCherry;lin-15(+)]*,

*lin-15(n765ts);gtl-2(tm1463);tgEx143[Psulp-4::mCherry;lin-15(+)]*,

*gtl-2(tm1463);tgEx133[Pgtl-2::gtl-2cDNA::gfp;Pgtl-2::mCherry]*,

*gtl-2(tm1463);tgEx135[Psulp-4::gtl-2cDNA::gfp;Psulp-4::mCherry]*,

*gtl-2(tm1463);tgEx124[Pgtl-2::gtl-2cDNA;sur-5::gfp]*,

*gtl-2(tm1463);tgEx131[Pgtl-2::gtl-1cDNA;sur-5::gfp]*,

*gtl-2(tm1463);tgEx140[Pgtl-2::gon-2cDNA;sur-5::gfp]*.

Transgenic strains were generated by injecting the mixed DNA of an expression plasmid (100 ng/µl) and a genetic marker plasmid (100 ng/µl) into young adult worms as described [34]. To test rescue of the *tm1463* allele by the *gtl-2* cDNA driven by *Pgtl-2*, at least three independent extrachromosomal lines were analyzed.

### Measurement of Trace Elements

Strains analyzed by ICP-MS were grown on NGM-lite plates. In each case, animals were rinsed off in HPEG (water with 0.1% PEG 8000), then washed twice prior to drying. Samples were analyzed by the Dartmouth ICPMS facility, as described previously [9]. Although worms analyzed by ICPMS typically contained some *E. coli* in the gut, the contribution of unabsorbed cations within the lumenal fluid to overall levels is likely to be minimal.

### Growth Assays

Each growth assay was performed as previously described [9]. Before starting each assay, animals were kept on electrolyte-free plates (see *C. elegans* strains section) for at least two generations at 20°C, except *gtl-1(tg113)* mutant animals. The *gtl-1* mutants were maintained on conventional NGM-lite plates to avoid growth defects [9]. About 50 eggs were collected and transferred to each assay plate. All strains were grown at 25°C and the number of adult animals was determined every 12 or 24 hours. All data for transgenic lines were corrected for the number of progeny that lost the extrachromosomal array.

### RNAi Experiments

For the data reported in Figure S2, feeding RNAi was performed as described by Kamath et al. [35], using one clone that contained a portion of the *gtl-1* cDNA(656 bp) and another that contained GFP (as control). We used NGM plates containing 25 µM carbenicillin instead of ampicllin, and 2 mM IPTG was used for induction. Other conditions were the same as in our conventional growth assays.

### Microscopy

Worms were immobilized with 0.03% (w/v) NaN_3_, and then mounted on 2% agar pads under coverslips. For Figure 2, images were taken using a Zeiss Axioskop 2, using a ccd (charge-coupled device) camera (1300; Micromax) and MetaMorph software (version 7.1; MDS Analytical Technologies). The fluorescence image was deconvoluted using AutoDeblur/AutoVisualize software (version 1.4.1.; Media Cybernetics). For Figure 3A and S1, images were taken using a color ccd camera (FLOVEL Inc.). Image brightness and contrast were adjusted using Photoshop CS3 software (Adobe).

### Embryonic Cell Culture

Primary cultured cells were prepared from transgenic animals as described previously [36]. Eexcretory cells were identified in primary culture based on mCherry expression.

### Electrophysiology

Whole-cell currents were recorded as described previously [9], [22]. Pipette electrodes were made from 1/0.58 OD/ID (mm) borosilicate glass capillary 1B100F-4 (World Precision Instruments) using a P-97 micropipette puller (Sutter Instruments). The electrodes had a resistance of 15–20 M ohm when filled with the standard pipette solution. The membrane potential was clamped at 0 mV and a 1–2 min period was allowed after rupture of the membrane to equilibrate the cell interior with pipette solution. Current through the electrode was recorded by 700A amplifier (Axon Instruments) after filtering at 5 kHz.

All data are corrected for the liquid junction potential of the pipette solution relative to Ringer's in the bath (+15 mV) and for leak currents collected in the standard extracellular solution containing 100 µM LaCl_3_, which completely inhibits currents (see Fig. 3D).

The standard pipette solution contained the following components (in mM) 147 sodium gluconate, 0.6 CaCl_2_, 1 MgCl_2_, 10 EGTA, 10 HEPES, 2 Na+ATP, 0.5 Na+GTP. The pH was adjusted to 7.2 with CsOH, and osmolarity was adjusted to 325 mOsm. The standard extracellular Ringer's solution contained (in mM): 145 NaCl, 1 CaCl_2_, 5 MgCl_2_, 10 Hepes, 20 Glucose, 24 sucrose. The pH was adjusted to 7.2 with NaOH, and osmolarity was adjusted to 345 mOsm. Voltage steps from −100 to +100 mV lasting 400 ms were applied every 1 sec from a holding potential of 0 mV. Averaged results are presented as the mean value ± SEM. All curve fitting was done by least-squares methods.

## Supporting Information

Figure S1 Subcellular l socalization of GTL-2. Top panel, DIC low magnification. Middle panel, a merged image of GFP and mCherry signal; low magnification. Bottom panel, a high magnification image of the canal. The imaged animal was of genotype: gtl-2(tm1463);tgEx133[Pgtl-2::gtl-2cDNA::gfp;Pgtl-2::mCherry]. Faint green fluorescence and particles were detected from auto-fluorescent granules in the intestine.
(0.87 MB TIF)

Figure S2 Effect of gtl-1(RNAi) on growth rates of wild type and gtl-2(tm1463)) mutant animals. RNAi was performed as described in Materials and Methods. When animals are grown on 0 mM Mg2+ plates gtl-1(RNAi) inhibits the growth rate of both wild type and gtl-2(tm1463) mutant animals, but wild type animals are much more severely affected. In wild type animals, 5 mM Mg2+, almost completely suppresses the growth inhibitory effect of gtl-1(RNAi). In gtl-2(tm1463) mutants, gtl-1(RNAi) efficiently rescues the growth arrest phenotype induced by 5 mM Mg2+.
(0.10 MB TIF)

Table S1 Trace elements measured in wild type and gtl-1 gtl-2 double mutants in response to different Mg2+ concentrations.
(0.05 MB DOC)
