# Supplementary figures and images for: Magnesium Excretion in C. elegans Requires the Activity of the GTL-2 TRPM Channel
Source: PLoS One. 2010 Mar 8;5(3):e9589. doi: 10.1371/journal.pone.0009589 (PMC2833210; doi:10.1371/journal.pone.0009589)

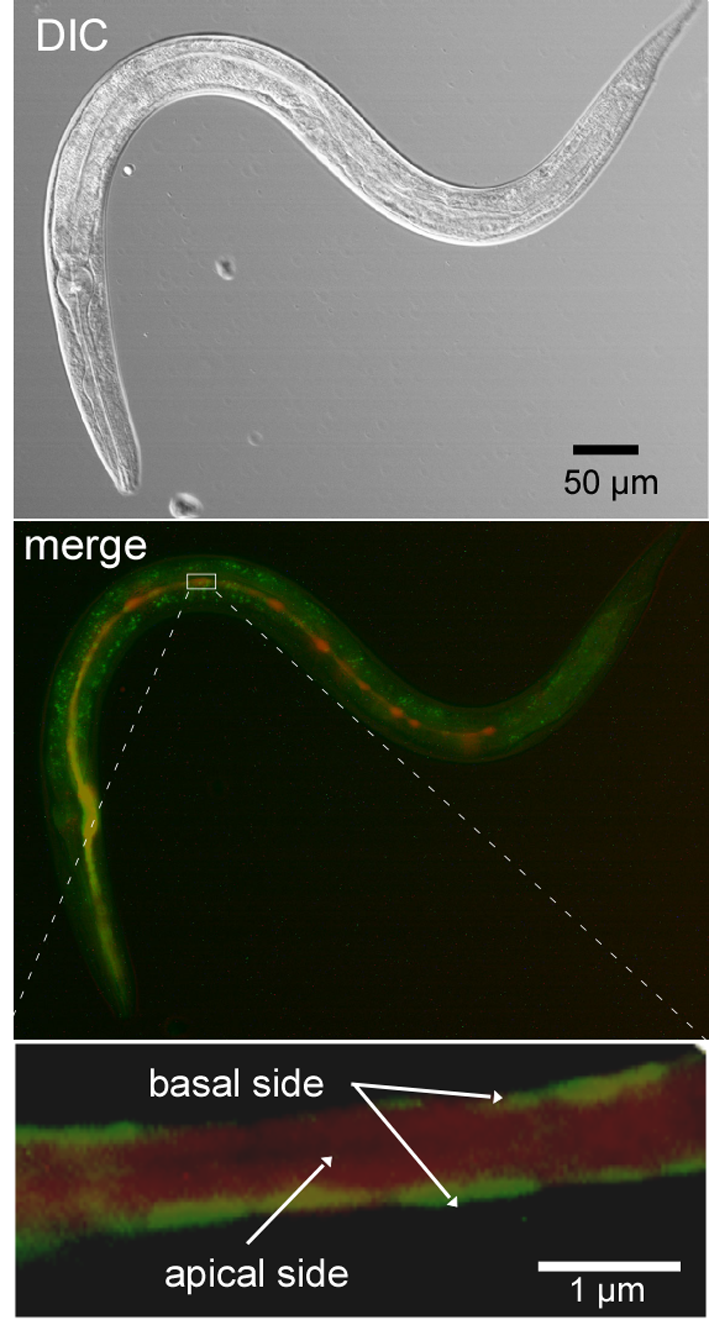

Supplement: Figure S1 — Subcellular l socalization of GTL-2. Top panel, DIC low magnification. Middle panel, a merged image of GFP and mCherry signal; low magnification. Bottom panel, a high magnification image of the canal. The imaged animal was of genotype: gtl-2(tm1463);tgEx133[Pgtl-2::gtl-2cDNA::gfp;Pgtl-2::mCherry]. Faint green fluorescence and particles were detected from auto-fluorescent granules in the intestine. (0.87 MB TIF) [file pone.0009589.s001.tif]

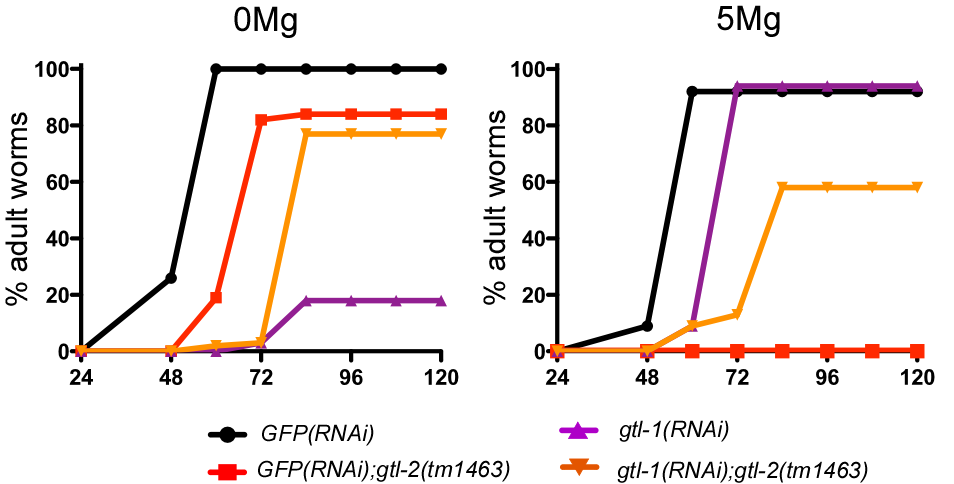

Supplement: Figure S2 — Effect of gtl-1(RNAi) on growth rates of wild type and gtl-2(tm1463)) mutant animals. RNAi was performed as described in Materials and Methods. When animals are grown on 0 mM Mg2+ plates gtl-1(RNAi) inhibits the growth rate of both wild type and gtl-2(tm1463) mutant animals, but wild type animals are much more severely affected. In wild type animals, 5 mM Mg2+, almost completely suppresses the growth inhibitory effect of gtl-1(RNAi). In gtl-2(tm1463) mutants, gtl-1(RNAi) efficiently rescues the growth arrest phenotype induced by 5 mM Mg2+. (0.10 MB TIF) [file pone.0009589.s002.tif]
