# Supplementary material for: Magnesium Excretion in C. elegans Requires the Activity of the GTL-2 TRPM Channel
Source: PLoS One. 2010 Mar 8;5(3):e9589. doi: 10.1371/journal.pone.0009589 (PMC2833210; doi:10.1371/journal.pone.0009589)
Supplement: Table S1 — Trace elements measured in wild type and gtl-1 gtl-2 double mutants in response to different Mg2+ concentrations. (0.05 MB DOC) [file pone.0009589.s003.doc]

| **Genotype** | **Mg supplementation level (mM)** | **Mg (μg/g)** | **K (μg/g)** | **Ca (μg/g)** |
| --- | --- | --- | --- | --- |
| Wild type | 0 | 2134 | 15080 | 2902 |
| Wild type | 1 | 2147 | 16224 | 2043 |
| Wild type | 5 | 2291 | 17487 | 1408 |
| *gtl-1(dx153) gtl-2(tm1463)* | 0 | 1518 | 15321 | 4629 |
| *gtl-1(dx153) gtl-2(tm1463)* | 1 | 1649 | 15668 | 3357 |
| *gtl-1(dx153) gtl-2(tm1463)* | 5 | 1913 | 16672 | 2216 |
| *gtl-1(dx170) gtl-2(tm1463)* | 0 | 1553 | 15192 | 5265 |
| *gtl-1(dx170) gtl-2(tm1463)* | 1 | 1574 | 15172 | 2894 |
| *gtl-1(dx170) gtl-2(tm1463)* | 5 | 4571 | 17876 | 2948 |
| *gtl-1(dx171) gtl-2(tm1463)* | 0 | 1347 | 14196 | 3944 |
| *gtl-1(dx171) gtl-2(tm1463)* | 1 | 1651 | 15441 | 2813 |
| *gtl-1(dx171) gtl-2(tm1463)* | 5 | 1766 | 14675 | 2200 |

**Table S1. Trace elements measured in wild type and *gtl-1 gtl-2* double mutants in response to different Mg^2+^ concentrations.** The reduction in Ca^2+^ levels in response to increased Mg^2+^ is probably due to inhibition of GON-2 in the intestinal cells.
